# Independent prognostic role of PD-L1 expression in patients with esophageal squamous cell carcinoma

**DOI:** 10.18632/oncotarget.14174

**Published:** 2016-12-26

**Authors:** Dongxian Jiang, Qi Song, Haixing Wang, Jie Huang, Hao Wang, Jun Hou, Xiaojing Li, Yifan Xu, Akesu Sujie, Haiying Zeng, Lijie Tan, Yingyong Hou

**Affiliations:** ^1^ Department of Pathology, Zhongshan Hospital, Fudan University, Shanghai 200032, P. R. China; ^2^ Department of thoracic surgery, Zhongshan Hospital, Fudan University, Shanghai 200032, P. R. China; ^3^ Department of Pathology, School of Basic Medical Sciences & Zhongshan Hospital, Fudan University, Shanghai 200032, P. R. China

**Keywords:** PD-L1 expression, clinical stage, lymph node metastasis, prognostic marker, ESCC

## Abstract

Accumulating evidence has shown that PD-L1 expression is associated with clinicopathological features in various human malignancies. We searched for correlations between PD-L1 expression and clinicopathological data in esophageal squamous cell carcinoma (ESCC) patients. PD-L1 expression in primary tumors from 278 patients was evaluated using immunohistochemistry (IHC) in ESCC tissue microarray. Survival curves were constructed by using the Kaplan-Meier method. Univariate and multivariate Cox proportional hazard regression models were performed to identify associations with outcome variables. Overall, tumoral PD-L1 expression (≥10%, 20% or 30% as cut-off value) was associated with favorable DFS and OS upon multivariate analysis. When the patients stratified into stage I-II (168, 60.4%) and stage III-IV (110, 39.6%), or with lymph node metastasis (133, 47.8%), the prognostic role was not consistent. In patients with stage I-II disease, tumoral PD-L1 expression (≥5%, 10%, 20% or 30%) was associated with better DFS and OS upon multivariate analysis. In patients without lymph node metastasis, tumoral PD-L1 expression (≥1%, 5%, 10%, 20%, or 30%) was associated with improved DFS and OS in univariate or multivariate analysis. However, PD-L1 expression was not correlated with prognosis in patients with stage III-IV disease or with lymph node metastasis. Our results for the first time showed the prognostic role of tumoral PD-L1 expression was variable in different stages and lymph node status of ESCC. Tumoral PD-L1 expression was independent favorable predictor in ESCC patients with Stage I-II disease or without lymph node metastasis, not in stage III-IV or lymph node metastasis.

## INTRODUCTION

Esophageal cancer (EC) remains one of the most fatal cancers worldwide with its incidence on the rise [1]. In 2015, EC had affected over 478,000 people across China and almost 375,000 had succumbed to this disease [2]. China alone accounts for more than 70% of EC worldwide, and 95% is esophageal squamous cell carcinoma (ESCC), which has poorer biological behavior than esophageal adenocarcinoma (EAC) [3]. Despite clinical advances in radiochemotherapy and target therapy, ESCC remains one of the leading causes of cancer-associated mortality, with 5-year survival rate no better than a mere 20%[1]. More recently, given the discovery of drugs able to interfere with specific immune checkpoints, cancer immunotherapy has entered into a new era and may be a novel strategy for the future ESCC treatment [4]. Considering the clinical benefit-risk of immunotherapy, useful biologic markers for assessing risk of ESCC progression are urgently needed.

Immune evasion has been recognized as an important factor of cancer progression [5]. Programmed death 1 (PD1, CD279) is important in regulating immune tolerance by inhibiting T or B cell activation. PD1 has two ligands: PD-Ligand1 (PD-L1, CD274, B7-H1) and PD-Ligand2 (PD-L2, B7-DC, CD273) [6]. PD-L1 has a much broader tissue distribution than PD-L2. PD-L1 is expressed not only on haematopoietic cells, but also on parenchymal cells. And PD-L1 appears to be the major ligand expressed in solid tumors and is frequently upregulated in human cancers [7]. There are two mechanisms of PD-L1 expression on tumor cells: 1) through genetic alterations or activation of certain signaling pathways (intrinsic immune resistance), 2) through an induced response to inflammatory signals (adaptive immune resistance) [8–10]. The inducible expression seems to be more common than the constitutive expression in most cancer. With this expression pattern, PD-L1 is adaptively induced as a consequence of the presence of tumor antigen-specific T cells, and these cancer cells expressed PD-L1 and turned off the specific cytotoxic immune response, which contributes to immune evasion and facilitates tumor growth [11, 12].

PD-L1 expression has been correlated with poor clinical outcomes in different cancers [13–15], such as melanoma, lung, breast, bladder, ovarian, salivary gland carcinomas, gastric cancer, kidney tumors as well as osteosarcoma [5, 16, 17]. However, other reports indicated a lack of association between PD-L1 expression and outcome [18, 19] or that PD-L1 expression was associated with an improved survival [14, 20]. In ESCC, PD-L1 expression has been very scarcely studied, only few prognostic studies, provided inconsistent conclusion. Chen et al recently suggested PD-L1 expression is a favorable indicator for ESCC prognosis [21], while other researchers found that PD-L1-positive ESCC patients had significantly poorer prognosis than the negative patients [22–24]. Therefore, further detailed analysis is needed to explore the prognostic significance of PD-L1 expression in ESCC.

Here, we have analyzed PD-L1 expression in 278 ESCCs using tissue microarrays, and compared its expression in different stage and different state of lymph node metastasis. We searched for correlations between PD-L1 expression and prognosis in ESCC, and intended to clarify the inconsistent conclusions in previous studies.

## RESULTS

### Patient characteristics

A total of 278 surgically resected FFPE primary ESCC sampled in TMAs could be assessed. The clinical and pathologic features of the study cohort are summarized in Table 1. Patients at diagnosis ranged in age from 37 to 83 years (median, 62.0 years) and were predominantly male (232 of 278, 83.5%). Of these, 58.3% were non-smokers and 41.4% were smokers. All 278 patients were treated by esophagectomy without neoadjuvant therapy. By anatomic site, 47.1% tumors were in the middle esophagus, 48.9% in the upper and lower area. On the basis of the AJCC Staging Manual (seventh edition), 163 (58.6%) cases were histologically graded as well to moderately differentiated, and 115 (41.4%) were poorly differentiated. Vessel and nerve involvement were identified in 51 (18.3%) and 73 (26.3%) tumors, respectively. Among these patients, 168 (60.4%) patients had AJCC pathologic stage I–II disease, and 110 (39.6%) stage III-IVa disease. Lymph node metastasis was identified in 133 (47.8%) patients (Table 1).

**Table 1:** Clinicopathological characteristics of the ESCC cohort

	All patients		Patients with Stage I-II disease		Patients without lymph node metastasis	
	n	%	n	%	n	%
Sex						
Female	46	16.5	35	20.8	29	20
Male	232	83.5	133	79.2	116	80
Age						
<60	116	41.7	62	36.9	51	35.2
≥60	162	58.3	106	63.1	94	64.8
Smoking						
No	162	58.3	110	65.5	95	65.5
Yes	115	41.4	57	33.9	49	33.8
Differentiation						
Well	7	2.5	4	2.4	4	2.8
Moderate	156	56.1	97	57.7	85	58.6
Poor	115	41.4	67	39.9	56	38.6
Invasive depth						
I	89	32	82	48.8	61	42.1
II	189	68	86	51.2	84	57.9
Vessel involvement						
No	227	81.7	157	93.5	137	94.5
Yes	51	18.3	11	6.5	8	5.5
Nerve involvement						
No	205	73.7	137	81.5	116	80
Yes	73	26.3	31	18.5	29	20
Lymph node metastasis						
No	145	52.2	135	80.4		
Yes	133	47.8	33	19.6		
Tumor site						
Upper	13	4.7	8	4.8	7	4.8
Middle	131	47.1	83	49.4	73	50.3
Low	123	44.2	66	39.3	54	37.2
Clinical stage						
I-II	168	60.4			135	93.1
III-Iva	110	39.6			10	6.9
tPD-L1 ≥1%						
No	137	49.3	88	52.4	73	50.3
Yes	141	50.7	80	47.6	72	49.7
tPD-L1 ≥5%						
No	153	55	96	57.1	78	53.8
Yes	125	45	72	42.9	67	46.2
tPD-L1 ≥10%						
No	180	64.7	116	69	95	65.5
Yes	98	35.3	52	31	50	34.5
tPD-L1 ≥20%						
No	199	71.6	124	73.8	102	70.3
Yes	79	28.4	44	26.2	43	29.7
tPD-L1 ≥30%						
No	221	79.5	134	79.8	113	77.9
Yes	57	20.5	34	20.2	32	22.1
tPD-L1 ≥50%						
No	233	83.8	142	84.5	121	83.4
Yes	45	16.2	26	15.5	24	16.6
sPD-L1 ≥1%						
No	162	58.3	97	57.7	88	60.7
Yes	116	41.7	71	42.3	57	39.3
sPD-L1 ≥5%						
No	171	61.5	103	61.3	91	62.8
Yes	107	38.5	65	38.7	54	37.2
sPD-L1 ≥10%						
No	235	84.5	142	84.5	121	83.4
Yes	43	15.5	26	15.5	24	16.6
sPD-L1 ≥20%						
No	236	84.9	142	84.5	121	83.4
Yes	42	15.1	26	15.5	24	16.6
sPD-L1 ≥30%						
No	249	89.6	150	89.3	128	88.3
Yes	29	10.4	18	10.7	17	11.7
sPD-L1 ≥50%						
No	260	93.5	157	93.5	134	92.4
Yes	18	6.5	11	6.5	11	7.6

Invasive depth I, tumor invasion confined to muscularis; Invasive depth II, tumor invasion beyond the muscularis.

tPD-L1, Tumoral PD-L1 expression; sPD-L1, Stromal PD-L1 expression.

### PD-L1 expression in ESCC specimens

**Figure 1:**
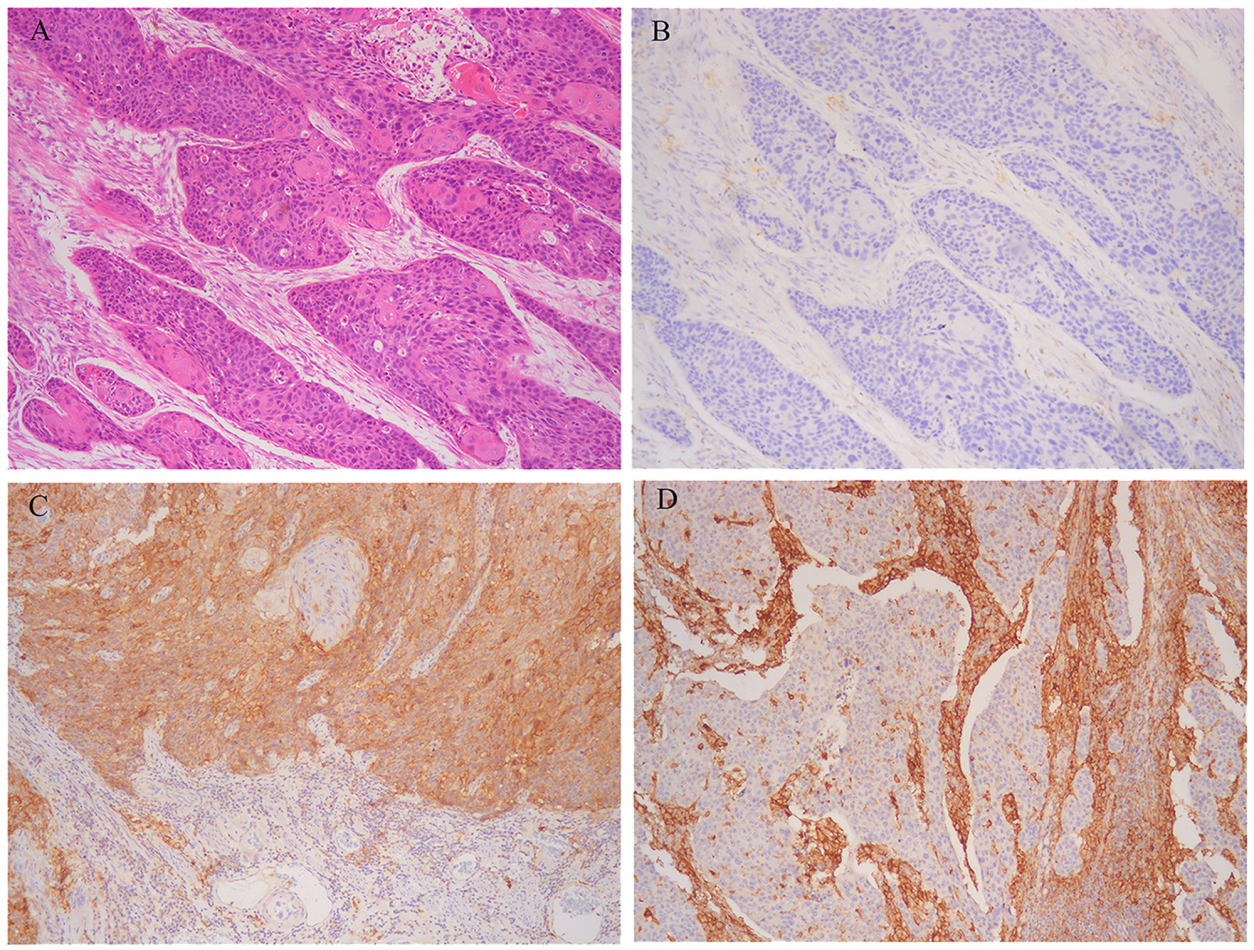
Representative microphotographs of sections from esophageal squamous cell carcinoma (ESCC) **A.** Moderately differentiated cancer cells. **B.** Programmed death-1 ligand-1 (PD-L1) expression neither in tumoral nor stromal cells. **C.** PD-L1 strong expression in tumoral cells. D. PD-L1 strong expression in stromal cells.

Among 278 cases analyzed, 50.7% of cases showed tumoral PD-L1 expression (defined as ≥1%), and 41.7% showed stromal immune cell expression (Figure 1). The levels of expression at ≥5% in each compartment are 45.0% and 38.5% respectively. As to 10%, 20%, 30% and 50% cut-points, 35.3%, 28.4%, 20.5% and 16.2% cases had membranous or cytoplasmic PD-L1 expression, and 15.5%, 15.1%, 10.4% and 6.5% cases had stromal expression. Tumoral PD-L1 expression was associated with stromal expression (*P*<0.05) (Supplementary Table 1-5).

### Survival analyses in the cohort of ESCC patients

The median follow-up period was 33 months (range 2-102 months). There was 162 disease progression documented. Mean and median times to disease free survival (DFS) were 38.8 and 28.0 months, respectively. A total of 163 patients (58.6%) died during the follow up, where 157 (56.5%) patients died of ESCC. Mean and median times to overall survival (OS) were 42.7 and 33.0 months, respectively.

The tumoral PD-L1 expression (≥5%, 10%, 20%, 30% or 50% as cut-off value) was associated with improved DFS (*P*=0.047, 0.039, 0.011, 0.002, or 0.05) and OS (*P*=0.031, 0.036, 0.010, 0.002 or 0.039), however, stromal expression was non-significant in the assessment of association with survival (Supplementary Table 6). Upon univariate analysis, stage, lymph node metastasis, vascular involvement, depth, and tumoral PD-L1 expression (≥10%, 20% or 30%) were associated with DFS, and on multivariate analysis, stage, lymph node metastasis and tumoral PD-L1 expression (≥10%, 20% or 30%) were associated with DFS (Table 2). Univariate analysis showed that stage, lymph node metastasis, vascular involvement, depth, and tumoral PD-L1 expression (≥5%, 10%, 20%, 30% or 50%) were associated with OS. Upon multivariate analysis, stage, lymph node metastasis, and tumoral PD-L1 expression (≥10%, 20%, 30% or 50%) were associated with OS (Table 3).

**Table 2:** Univariate and multivariate Cox regression analysis of clinicopathological and molecular features for disease free survival

Variable	All patients				Patients with Stage I-II disease					Patients with Stage III-IVa disease				Patients without lymph node metastasis				Patients with lymph node metastasis		
	P value	Hazard ratio (CI 95%)			P value	Hazard ratio (CI 95%)			P value		Hazard ratio (CI 95%)			P value	Hazard ratio (CI 95%)			P value	Hazard ratio (CI 95%)	
univariate analysis																			
Sex	0.498	1.159(0.756-1.775)			0.537	0.843(0.489-1.452)			0.939	1.029(0.497-2.129)			0.965	1.015(0.524-1.966)			0.994	1.002(0.571-1.759)	
Age	0.650	1.075(0.786-1.472)			0.267	1.321(0.808-2.159)			0.451	1.174(0.774-1.778)			0.555	1.185(0.674-2.085)			0.086	1.399(0.954-2.053)	
Smoking	0.252	1.198(0.879-1.633)			0.842	1.06(0.649-1.733)			0.598	0.895(0.591-1.354)			0.459	1.228(0.713-2.117)			0.407	0.852(0.583-1.245)	
Differentiation	0.132				0.178				0.791				0.374				0.422		
Moderate	0.289	0.535(0.169-0.698)			0.963	0(0-3.531E223)			0.532	1.458(0.448-4.749)			0.966	0(0-5.612E+243)			0.402	1.652(0.511-5.340)	
Poor	0.065	0.746(0.546-1.018)			0.063	0.645(0.406-1.024)			0.882	0.968(0.634-1.478)			0.161	0.685(0.403-1.163)			0.394	0.846(0.575-1.244)	
Invasive depth	0.001	1.885(1.309-2.713)			0.822	0.972(0.606-1.559)			0.047	3.222(1.016-10.222)			0.368	1.287(0.743-2.231)			0.011	1.934(1.162-3.221)	
Vessel involvement	<0.001	1.960(1.376-2.791)			0.555	1.339(0.5779-3.097)			0.54		1.141(0.748-1.741)			0.537	1.378(0.497-3.819)			0.338	1.214(0.817-1.806)	
Nerve involvement	0.168	1.270(0.904-1.785)			0.071	0.508(0.244-1.061)			0.16	1.354(0.887-2.067)			0.062	0.446(0.191-1.041)			0.016	1.628(1.093-2.425)	
Lymph node metastisis	<0.001	3.343(2.403-4.650)			<0.001	2.853(1.751-4.648)			0.728	0.879(0.425-1.819)									
Tumor site	0.091				0.014				0.092				0.022				0.146		
Middle	0.646	1.187(0.570-2.472)			0.941	1.047(0.313-3.500)			0.050	2.574(1.001-6.615)			0.963	1.036(0.237-4.536)			0.224	1.694(0.724-3.959)	
Low	0.029	1.427(1.038-1.964)			0.005	2.074(1.249-3.444)			0.172	1.347(0.879-2.064)			0.008	2.254(1.238-4.102)			0.076	1.426(0.964-2.109)	
Clinical stage	<0.001	3.323(2.421-4.561)											<0.001	4.546(2.123-9.736)			0.064	1.532(0.976-2.404)	
tPDL1 ≥1%	0.118	0.781(0.573-1.064)			0.043	0.611(0.379-0.985)			0.267	0.791(0.522-1.197)			0.03	0.548(0.318-0.944)			0.974	1.006(0.688-1.472)	
tPDL1≥5%	0.052	0.731(0.533-1.003)			0.009	0.514(0.311-0.850)			0.613	0.898(0.593-1.361)			0.028	0.536(0.308-0.935)			0.997	1.001(0.680-1.472)	
tPDL1≥10%	0.044	0.706(0.503-0.991)			0.005	0.424(0.232-0.773)			0.239	0.774(0.505-1.186)			0.019	0.465(0.245-0.881)			0.634	0.906(0.605-1.359)	
tPDL1 ≥20%	0.013	0.620(0.425-0.906)			0.003	0.344(0.171-0.692)			0.332	0.796(0.502-1.263)			0.006	0.347(0.164-0.735)			0.885	0.968(0.622-1.506)	
tPDL1 ≥30%	0.003	0.501(0.316-0.792)			0.005	0.300(0.130-0.692)			0.17	0.678(0.389-1.181)			0.01	0.297(0.118-0.744)			0.272	0.742(0.436-1.263)	
tPDL1 ≥50%	0.057	0.628(0.389-1.013)			0.065	0.455(0.197-1.051)			0.223	0.693(0.385-1.249)			0.098	0.459(0.183-1.153)			0.309	0.747(0.426-1.311)	
sPDL1 ≥1%	0.507	1.111(0.815-1.515)			0.107	1.463(0.921-2.324)			0.465	0.854(0.560-1.304)			0.115	1.532(0.901-2.604)			0.112	0.732(0.499-1.076)	
sPDL1 ≥5%	0.621	1.082(0.791-1.481)			0.215	1.343(0.843-2.140)			0.483	0.858(0.560-1.315)			0.108	1.547(0.909-2.634)			0.128	0.738(0.500-1.091)	
sPDL1 ≥10%	0.216	0.749(0.473-1.185)			0.348	0.716(0.356-1.440)			0.513	0.816(0.443-1.501)			0.357	0.689(0.311-1.523)			0.528	0.834(0.475-1.465)	
sPDL1 ≥20%	0.275	0.775(0.490-1.225)			0.348	0.716(0.356-1.440)			0.819	0.931(0.506-1.714)			0.357	0.689(0.311-1.523)			0.787	0.925(0.526-1.626)	
sPDL1 ≥30%	0.267	0.733(0.424-1.269)			0.378	0.686(0.297-1.584)			0.614	0.829(0.400-1.718)			0.436	0.694(0.276-1.741)			0.664	0.859(0.433-1.706)	
sPDL1 ≥50%	0.433	0.764(0.390-1.497)			0.633	0.782(0.285-2.145)			0.502	0.734(0.297-1.812)			0.824	0.891(0.322-2.467)			0.634	0.803(0.327-1.976)	
Multivariate analysis																			
Clinical stage	0.020	1.833(1.100-3.053)			-				-				<0.001	4.941(2.199-11.106)			-		
Lymph node metastasis	0.004	2.036(1.255-3.304)			<0.001	2.538(1.550-4.157)			-				-				-		
Vessel involvement	0.571	1.115(0.765-1.627)			-				-				-				-		
Nerve involvement	-				-				-				-				0.131	1.382(0.908-2.105)	
Invasive depth	0.302	1.245(0.821-1.886)			-				-				-				0.051	1.709(0.997-2.932)	
Tumor site	-				0.008				-				0.067				-		
Middle					0.791	1.178(0.350-3.961)							0.967	0.969(0.220-4.264)					
Low					0.002	2.218(1.331-3.694)							0.027	1.978(1.080-3.623)					
tPDL1 ≥1%	-				0.134	0.691(0.426-1.121)			-				0.012	0.480(0.270-0.852)			-		
tPDL1 ≥5%	0.116	0.773(0.561-1.066)			0.030	0.571(0.344-0.948)			-				0.008	0.453(0.253-0.813)			-		
tPDL1 ≥10%	0.028	0.678(0.480-0.959)			0.042	0.534(0.291-0.978)			-				0.008	0.386(0.192-0.776)			-		
tPDL1 ≥20%	0.028	0.649(0.442-0.954)			0.019	0.428(0.211-0.868)			-				0.002	0.286(0.130-0.632)			-		
tPDL1 ≥30%	0.006	0.524(0.331-0.831)			0.021	0.372(0.161-0.862)				-				0.030	0.355(0.140-0.902)			-		

**Table 3:** Univariate and multivariate Cox regression analysis of clinicopathological and molecular features for esophageal cancer-specific survival

	All patients				Patients with Stage I-II disease					Patients with Stage III-IVa disease				Patients without lymph node metastasis				Patients with lymph node metastasis		
Variable	P value	Hazard ratio (CI 95%)			P value	Hazard ratio (CI 95%)				P value	Hazard ratio (CI 95%)			P value	Hazard ratio (CI 95%)			P value	Hazard ratio (CI 95%)	
Univariate analysis																				
Sex	0.471	1.174(0.760-1.813)			0.400	0.790(0.457-1.367)				0.735	1.143(0.528-2.476)			0.911	0.981(0.703-1.369)			0.989	1.002(0.749-1.340)	
Age	0.626	1.082(0.788-1.488)			0.145	1.459(0.878-2.423)				0.516	1.149(0.755-1.748)			0.360	1.317(0.730-2.377)			0.083	1.407(0.957-2.069)	
Smoking	0.193	1.232(0.900-1.686)			0.830	1.055(0.646-1.724)				0.592	0.892(0.586-1.357)			0.418	1.260(0.720-2.203)			0.403	0.849(0.578-1.246)	
Differentiation	0.200				0.280				0.571				0.523				0.326		
Moderate	0.333	0.565(0.178-1.794)			0.963	0(0-1.967E222)				0.303	1.865(0.570-6.102)			0.967	0(0-8.477E+244)			0.227	2.067(0.637-6.708)	
Poor	0.102	0.768(0.560-1.054)			0.111	0.681(0.424-1.092)				0.971	0.992(0.647-1.521)			0.256	0.729(0.422-1.257)			0.506	0.876(0.594-1.293)	
Invasive depth	<0.001	2.051(1.407-2.989)			0.959	0.988(0.616-1.584)				0.048	4.121(1.012-16.783)			0.250	1.399(0.790-2.477)			0.011	1.960(1.164-3.301)	
Vessel involvement	<0.001	2.083(1.456-2.982)			0.438	1.394(0.602-3.226)				0.469	1.171(0.763-1.797)			0.405	1.544(0.556-4.289)			0.321	1.226(0.820-1.833)	
Nerve involvement	0.069	1.378(0.976-1.945)			0.147	0.580(0.277-1.212)				0.125	1.397(0.912-2.139)			0.125	0.513(0.219-1.202)			0.016	1.635(1.095-2.442)	
Lymph node metastisis	<0.001	3.646(2.604-5.106)			<0.001	3.147(1.922-5.155)				0.862	0.937(0.452-1.943)									
Tumor site	0.192			0.019			0.112			0.024			0.235	
Middle	0.640	1.192(0.571-2.486)			0.895	1.086(0.322-3.655)				0.046	2.604(1.015-6.677)			0.787	1.228(0.277-5.447)			0.289	1.584(0.677-3.704)	
Low	0.069	1.351(0.977-1.869)			0.006	2.077(1.228-3.513)				0.286	1.266(0.821-1.952)			0.008	2.359(1.253-4.440)			0.124	1.364(0.918-2.025)	
Clinical stage	<0.001	3.568(2.586-4.924)											<0.001	5.021(2.336-10.795)			0.067	1.525(0.970-2.396)	
tPDL1 ≥1%	0.074	0.750(0.547-1.029)			0.028	0.577(0.353-0.943)				0.310	0.805(0.530-1.224)			0.023	0.519(0.295-0.913)			0.868	0.968(0.659-1.422)	
tPDL1≥5%	0.034	0.706(0.511-0.975)			0.005	0.471(0.280-0.793)				0.755	0.935(0.613-1.426)			0.018	0.497(0.278-0.887)			0.945	0.986(0.667-1.459)	
tPDL1≥10%	0.041	0.695(0.490-0.984)			0.005	0.409(0.219-0.762)				0.290	0.790(0.511-1.222)			0.020	0.453(0.233-0.882)			0.619	0.901(0.596-1.361)	
tPDL1 ≥20%	0.012	0.608(0.412-0.897)			0.004	0.362(0.179-0.729)				0.284	0.771(0.479-1.241)			0.011	0.374(0.176-0.794)			0.683	0.909(0.576-1.435)	
tPDL1 ≥30%	0.002	0.475(0.294-0.767)			0.008	0.321(0.139-0.743)				0.095	0.605(0.335-1.091)			0.016	0.324(0.129-0.814)			0.138	0.653(0.372-1.147)	
tPDL1 ≥50%	0.044	0.597(0.360-0.987)			0.101	0.496(0.214-1.147)				1.124	0.608(0.323-1.146)			0.151	0.508(0.202-1.280)			0.151	0.643(0.352-1.175)	
sPDL1 ≥1%	0.686	1.067(0.779-1.463)			0.110	1.471(0.917-2.360)				0.173	0.743(0.484-1.140)			0.137	1.512(0.877-2.608)			0.059	0.688(0.466-1.015)	
sPDL1 ≥5%	0.777	1.047(0.761-1.440)			0.213	1.352(0.841-2.175)				0.207	0.757(0.491-1.167)			0.125	1.534(0.888-2.649)			0.082	0.704(0.474-1.045)	
sPDL1 ≥10%	0.141	0.703(0.439-1.124)			0.257	0.653(0.312-1.365)				0.240	0.694(0.377-1.278)			0.231	0.594(0.254-1.392)			0.425	0.795(0.452-1.397)	
sPDL1 ≥20%	0.175	0.723(0.452-1.156)			0.257	0.653(0.312-1.365)				0.386	0.763(0.414-1.406)			0.231	0.594(0.254-1.392)			0.601	0.860(0.489-1.512)	
sPDL1 ≥30%	0.274	0.736(0.425-1.275)			0.451	0.725(0.313-1.675)				0.386	0.724(0.350-1.501)			0.547	0.753(0.299-1.895)			0.430	0.759(0.382-1.506)	
sPDL1 ≥50%	0.410	0.754(0.384-1.478)			0.745	0.846(0.308-2.323)				0.270	0.601(0.243-1.485)			0.993	1.004(0.362-2.788)			0.370	0.662(0.269-1.630)	
Multivariate analysis																			
Clinical stage	0.029	1.771(1.059-2.963)			-				-				<0.001	4.645(2.108-10.236)			-		
Lymph node metastasis	0.001	2.238(1.373-3.647)			<0.001	2.800(1.702-4.607)				-				-				-		
Vessel involvement	0.517	1.135(0.774-1.665)			-				-				-				-		
Nerve involvement	-				-				-				-				0.133	1.384(0.906-2.114)	
Invasive depth	0.182	1.341(0.872-2.063)			-				-				-				0.052	1.727(0.995-2.999)	
Tumor site	-				0.008				-				0.098				-		
Middle					0.756	1.214(0.358-4.122)								0.795	1.219(0.274-5.416)			-		
Low					0.002	2.272(1.338-3.857)								0.035	2.000(1.052-3.804)			-		
tPDL1 ≥1%	-				0.086	0.646(0.392-1.064)				-				0.016	0.491(0.275-0.873)			-		
tPDL1≥5%	0.102	0.761(0.549-1.055)			0.014	0.516(0.305-0.874)				-				0.008	0.448(0.248-0.809)			-		
tPDL1≥10%	0.033	0.680(0.477-0.969)			0.040	0.518(0.276-0.969)				-				0.012	0.406(0.202-0.817)			-		
tPDL1 ≥20%	0.030	0.647(0.437-0.959)			0.027	0.450(0.222-0.915)				-				0.005	0.331(0.152-0.722)			-		
tPDL1 ≥30%	0.004	0.493(0.305-0.798)			0.033	0.401(0.173-0.929)				-				0.052	0.397(0.156-1.010)			-		
tPDL1 ≥50%	0.032	0.574(0.346-0.953)			0.096	0.490(0.212-1.134)				-				-				-		

**Figure 2:**
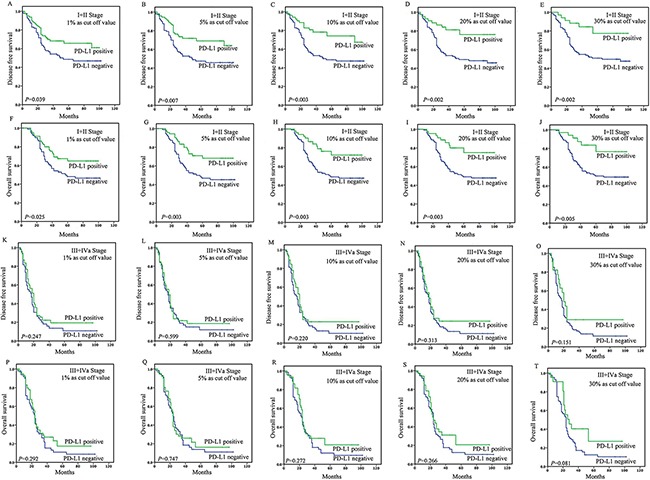
Survival analyses based on clinical stage of esophageal squamous cell carcinoma (ESCC) In patients with stage I-II disease, the tumoral PD-L1 expression (≥1%, 5%, 10%, 20%, or 30% as cut-off value) was associated with improved DFS (P=0.039, 0.007, 0.003, 0.002, or 0.002) A-E. and OS (P=0.025, 0.003, 0.003, 0.003 or 0.005) F-J. However, in patients with stage III-IV disease, the tumoral PD-L1 expression (≥1%, 5%, 10%, 20%, or 30% as cut-off value) was not associated with DFS (P=0.247, 0.599, 0.220, 0.313, or 0.151) K-O. and OS (P=0.292, 0.747, 0.272, 0.266, or 0.081) P-T.

In patients with stage I-II disease, the tumoral PD-L1 expression (≥1%, 5%, 10%, 20%, or 30% as cut-off value) was associated with improved DFS (*P*=0.039, 0.007, 0.003, 0.002, or 0.002) and OS (*P*=0.025, 0.003, 0.003, 0.003 or 0.005) (Figure 2), while stromal expression was non-significant in the assessment of association with survival (Supplementary Table 6). Upon univariate analysis, lymph node metastasis, site, and tumoral PD-L1 expression (≥1%, 5%, 10%, 20%, or 30%) were associated with DFS and OS, and on multivariate analysis, lymph node metastasis, site, and tumoral PD-L1 expression (≥5%, 10%, 20%, or 30%) were associated with DFS and OS (Table 2 and 3).

In patients with stage III-IV ESCC, the tumoral PD-L1 expression (≥1%, 5%, 10%, 20%, 30% or 50% as cut-off value) was not associated with DFS (*P*=0.247, 0.599, 0.220, 0.313, 0.151, or 0.202) and OS (*P*=0.292, 0.747, 0.272, 0.266, 0.081 or 0.108) (Figure 2). And stromal expression was also non-significant in the assessment of association with survival (Supplementary Table 6). Upon univariate analysis, only invasive depth was associated with DFS and OS (HR=3.222 and 4.121, *P*=0.047 and 0.048) (Table 2 and 3).

### Survival analyses based on lymph node status

**Figure 3:**
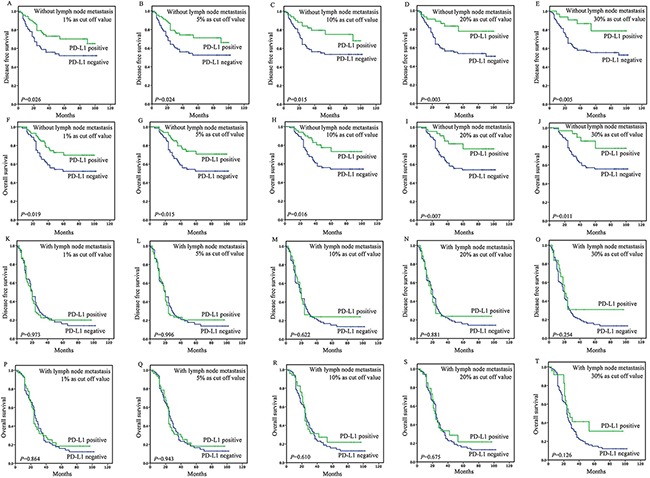
Survival analyses based on lymph node status of esophageal squamous cell carcinoma (ESCC) In patients without lymph node metastasis, the tumoral PD-L1 expression (≥1%, 5%, 10%, 20%, or 30% as cut-off value) was associated with improved DFS (P=0.026, 0.024, 0.015, 0.003, or 0.005) **A-E.** and OS (P=0.019, 0.015, 0.016, 0.007 or 0.011) **F-J.** However, in patients with lymph node metastasis, the tumoral PD-L1 expression (≥1%, 5%, 10%, 20%or 30% as cut-off value) was not associated with DFS (P=0.973, 0.996, 0.622, 0.881 or 0.254) **K-O.** and OS (P=0.864, 0.943, 0.610, 0.675, or 0.126) P-T.

In patients without lymph node metastasis, the tumoral PD-L1 expression (≥1%, 5%, 10%, 20%, or 30% as cut-off value) was associated with improved DFS (*P*=0.026, 0.024, 0.015, 0.003, or 0.005) and OS (*P*=0.019, 0.015, 0.016, 0.007 or 0.011) (Figure 3), however, stromal expression was non-significant in the assessment of association with survival (Supplementary Table 6). Upon univariate analysis, stage, site and tumoral PD-L1 expression (≥1%, 5%, 10%, 20%, or 30%) were associated with DFS and OS, and on multivariate analysis, stage and tumoral PD-L1 expression (≥1%, 5%, 10%, 20%, or 30%) were associated with DFS and OS (Table 2 and 3).

In patients with lymph node metastasis, the tumoral PD-L1 expression (≥1%, 5%, 10%, 20%, 30% or 50% as cut-off value) was not associated with DFS (*P*=0.973, 0.996, 0.622, 0.881, 0.254, or 0.291) and OS (*P*=0.864, 0.943, 0.610, 0.675, 0.126 or 0.138) (Figure 3). And stromal expression was also non-significant in the assessment of association with survival (Supplementary Table 6). Upon univariate analysis, invasive depth and nerve involvement were associated with DFS (HR=1.934 and 1.628, *P*=0.011 and 0.016) and OS (HR=1.960 and 1.635, *P*=0.011 and 0.016), and on multivariate analysis, no independent prognostic factor was identified (Table 2 and 3).

## DISCUSSION

The PD1-PD-L1 pathway has been important factors in cancer progression [25]. PD-L1 is expressed on a number of tumors, where it is believed to play a major role in immune suppression within tumor microenviroment (TME). However, there have been conflicting results in the literature as to whether PD-L1 expression is a favorable or adverse prognostic variable [17, 20, 26]. It is likely that the cohorts of patients, methods of assessment, definitions of cut-off value may account for these results.

Firstly, in previous studies, most of researchers put the collected patients as a whole, with no consideration of clinical stage. Here, we first divided our patients into different groups according to their clinical stage or the state of lymph node metastasis, to evaluate the prognostic significance of PD-L1 expression in ESCC. We found in ESCC patients with earlier stage (stage I-II or lymph node-negative), PD-L1 expression was associated with a significant better prognosis, while a lack of association between PD-L1 expression and outcome in patients with later stage (stage III-IV or lymph node-positive). This suggested the prognostic significance of PD-L1 expression was conditioned, only limited in some stages, not in all stages of ESCC.

Some researches also found the prognostic significance of biomarkers might differ in patients with different stages. For example, the prognostic impact of defective DNA mismatch repair (dMMR) appears to be stronger in earlier stage colorectal cancer (CRC) (stage II) than in later CRC (lymph node-positive or stage III) [27]. T-bet expression was associated with a significant better survival in CRC patients with stage I or II, however, it tended to be associated with a significant worse survival in patients with stage III or IV [28]. In NSCLC, PD-L1 expression appears to be a favorable prognostic factor in early stage disease, and the results may differ in advanced stage disease [20]. Therefore when facing with conflicting conclusions in prognostic analysis, it's needed to be further analyzed in patients with different stage or lymph node state, in order to speculate the underlying reasons for the results.

Secondly, to evaluate the PD-L1 expression at different level (mRNA level or protein level) in former studies, may contribute to the conflicting results, notably regarding the prognostic value. For example, in breast cancer, PD-L1 mRNA expression measured using an ISH assay was associated with a long recurrence-free survival [29], whereas protein expression measured using IHC was associated with a poor survival [30]. The mRNA-based method quantifies expression level of both tumor cells and non-tumor cells, including immune infiltrating cells, while IHC has the advantage of visualization and localization of the signal and identification of the different labeled populations (tumor and stromal expression) [31]. Our analysis at the protein level and based on tissue microarrays allowed us to avoid the limitations of mRNA-based methods and to work on a very large series of samples. We found tumoral expression was associated with better prognosis, while stromal expression had no prognostic value. Our results first reported different localization of PD-L1 expression had different value in ESCC, which was observed in other tumors [14]. For example, in breast cancer, cytoplasmic expression of PD-L1 was associated with improved patient survival for breast cancer-specific death, however, stromal expression fell short of significance for breast cancer-specific death [14]. This emphasizes the advantage of evaluating PD-L1 expression using IHC method.

Thirdly, the determination of cut-off values for the percentage of stained cells was difficult, and the absence of optimal positivity cut-off might be correlated with divergent results in previous studies. The results of the CheckMate 057 study have recently shown that PD-L1 expression (cut-off point 1%) significantly correlated with ORR (overall response rates), PFS (progression free survival) and OS, in pre-treated NSCLC patients [32]. In the prognostic analysis, different IHC cut-off points, ranging from 1% to 50%, have been used to define the PD-L1 positivity in tumor specimens [16, 21, 33]. Here, our work demonstrated the tumoral and stromal PD-L1 expression at different cut-off level of 1%, 5%, 10%, 20%, 30% and 50% to explore the best cut-off in prognostic analysis. Univariate and multivariate analysis found tumoral PD-L1 expression at the lowest level (cut-off point 1% or 5%) was independent prognostic factor among patients without lymph node metastasis or with Stage I-II disease. This suggests the cut off value should be down regulated to 1% or 5% in earlier stage ESCC.

PD-L1 expression was associated with a better outcome in earlier stage of ESCC, which has also been observed in NSCLC [34], pulmonary lymphoepithelioma-like carcinoma [35],colorectal cancer [36], breast cancer [14], and melanoma [8]. The biology of the association between PD-L1 expression and better outcome is not well understood. A potential explanation is a mixed immune cell infiltrate, which reflects a partially dysbalanced local cellular immune response and contributes to antitumor immune control. Some studies have suggested that PD-L1 could also provide positive signal through an unknown receptor other than PD-1, resulting in T-cell proliferation and induction of certain cytokines such as interleukin-10 and IFN-γ [6]. The systemic immunologic environment may affect the tumor growth to varying degrees. Further studies are required to clarify the molecular mechanisms responsible for regulation of PD-L1 expression.

In summary, we demonstrated tumoral PD-L1 expression at large-range levels (IHC cut-off from1% to 30%) was independent prognostic factor, associated with good prognosis in earlier stage of ESCC (with Stage I-II disease or without lymph node metastasis), not in later stage of ESCC (with Stage III-IV disease or with lymph node metastasis). Notably, the provocative observation indicated the prognostic significance of PD-L1 expression might limit in earlier stage of ESCC. Complex molecular mechanisms might involve in the biology of later stage ESCC, which reduce the biological role of PD-L1 expression. This also suggest the clinical significance of biomarkers should be analyzed according to different clinical stage in the clinical practice.

## MATERIALS AND METHODS

### Patients and specimens

This retrospective study was conducted in a cohort of 278 ESCC patients, who underwent curative resection (TNM stage: I-IVa) without preoperative chemotherapy at the Department of Thoracic Surgery, Zhongshan Hospital affiliated to Fudan University, Shanghai, China, between 2007 and 2010. Our research was approved by the local Ethics Committee of Zhongshan Hospital and was conducted in accordance with the ethical principles stated in the most recent version of the Declaration of Helsinki. All patients were pathologically staged according to the TNM classification system of the American Joint Committee for Cancer.

### Tissue microarrays

Tissue microarrays (TMA) were constructed as previously described [37]. Hematoxylin and eosin (HE)-stained slides were reviewed and the representative areas of interest with a high density of tumor cells were circled. The corresponding regions were marked on archival formalin-fixed, paraffin-embedded (FFPE) tissue blocks. The representative areas (2 mm wide and 6 mm long) was extracted, with at least three cores taken from different regions of the tumor, and then vertically planted into the recipient block one by one according to the corresponding location. The planting surface was aggregated on the aggregation instrument. An array was constructed with a maximum of 70 cores and a stomach core was used as an orientation marker.

### Immunohistochemical (IHC)

IHC labeling was performed on 4-mm-thick unstained sections. Slides were deparaffinized with serial xylene treatments and subjected to antigen retrieval using heated citrate solution (pH 9.0) at 100°C for 10 minutes. Immunolabeling for PD-L1 (SP142 Rabbit monoclonal, dilution 1:300, OriGene Technologies, Maryland, USA) was performed on the automated Ventana Benchmark XT system using the biotin free Ventana OptiView DAB IHC Detection Kit (Ventana Medical Systems, Tucson, AZ). For assessment of staining, slides were scanned with the ScanScope System (Aperio, CA) and viewed with ImageScope (Aperio).

### Pathological assessment of PD-L1 expression

PD-L1 IHC was evaluated in consensus viewing by two experienced pathologists, who were blinded to the clinical data, independently analyzed the PD-L1 expression. The results were evaluated according to the percentage of the stained cells and the intensity of the IHC signal intensity. Scoring was assessed in both the tumoral and stromal compartments: tumoral membranous or cytoplasmic expression and stromal immune cell compartments [14]. Similar to many studies in other cancers [38–40], tumors were classified as PD-L1-positive if there was ≥1% tumoral membranous or cytoplasmic PD-L1 expression, or ≥1% stromal PD-L1 expression. Because the most appropriate cut-off value for PD-L1 expression in ESCC remains unclear, analyses were also performed utilizing ≥5%, ≥10%, ≥20%, ≥30% and ≥50% cut-points for tumor PD-L1 positivity in each compartment, as the cut-offs were utilized in other study of PD-L1[41–43].

### Statistical analysis

The Chi-square test was also used for comparing clinicopathologic features between tumor groups. The date of last follow up was August 15, 2015. The time of DFS was defined as the time between diagnosis and local recurrence, distant metastasis, or death from ESCC. The time of OS was defined as the time between diagnosis and death from ESCC. Kaplan–Meier 5-year survival curves were generated and log-rank analyses were performed. Univariate Cox proportional hazard models were fitted to identify factors significantly correlated with DFS and OS. To assess whether the PD-L1 expression was an independent predictor of survival, a multivariate Cox model was constructed to adjust other clinicopathological characteristics that were significant in the univariate analyses.

All the statistical analyses were accomplished by the IBM SPSS statistics version 19.0 (SPSS Inc. Illinois, USA). Statistical significance was determined as p-values <0.05.

## SUPPLEMENTARY MATERIALS TABLES




