# Supplementary material for: Independent prognostic role of PD-L1 expression in patients with esophageal squamous cell carcinoma
Source: Oncotarget. 2016 Dec 26;8(5):8315–29. doi: 10.18632/oncotarget.14174 (PMC5352403; doi:10.18632/oncotarget.14174)
Supplement: Supplementary file 1 [file oncotarget-08-8315-s001.pdf]

Independent prognostic role of PD-L1expression in patients with esophageal squamous cell carcinoma

SUPPLEMENTARY TABLES

Supplementary Table 1: Correlation of PD-L1 expression with clinicopathological features in all ESCC patients

See Supplementary File 1.

Supplementary Table 2: Correlation of PD-L1 expression with clinicopathological features in ESCC patients with Stage I-II disease

See Supplementary File 1.

Supplementary Table 3: Correlation of PD-L1 expression with clinicopathological features in ESCC patients with Stage III-IV disease

See Supplementary File 1.

Supplementary Table 4: Correlation of PD-L1 expression with clinicopathological features in ESCC patients without lympha node metastasis

See Supplementary File 1.

Supplementary Table 5: Correlation of PD-L1 expression with clinicopathological features in ESCC patients with lympha node metastasis

See Supplementary File 1.

Supplementary Table 6: Kaplan–Meier survival analysis for disease free survival and esophageal cancer-specific survival

See Supplementary File 1.
