# Supplementary material for: Independent prognostic role of PD-L1 expression in patients with esophageal squamous cell carcinoma
Source: Oncotarget. 2016 Dec 26;8(5):8315–29. doi: 10.18632/oncotarget.14174 (PMC5352403; doi:10.18632/oncotarget.14174)
Supplement: Supplementary file 2 [file oncotarget-08-8315-s002.doc]

| Supplemental table 1 Correlation of PD-L1 expression with clinicopathological features in all ESCC patients | | | | | | | | | | | | | | | | | | |
| --- | --- | --- | --- | --- | --- | --- | --- | --- | --- | --- | --- | --- | --- | --- | --- | --- | --- | --- |
|  |  | tPDL1 ≥1% | |  | tPDL1 ≥5% | |  | tPDL1≥10% | |  | tPDL1 ≥20% | |  | tPDL1 ≥30% | |  | tPDL1 ≥50% | |
|  | N | % | *P* value |  | % | *P* value |  | % | *P* value |  | % | *P* value |  | % | *P* value |  | % | *P* value |
| Sex |  |  | 0.282 |  |  | 0.384 |  |  | 0.454 |  |  | 0.979 |  |  | 0.305 |  |  | 0.496 |
| Female | 46 | 43.5 |  |  | 39.1 |  |  | 30.4 |  |  | 28.3 |  |  | 26.1 |  |  | 19.6 |  |
| Male | 232 | 52.2 |  |  | 46.1 |  |  | 36.2 |  |  | 28.4 |  |  | 19.4 |  |  | 15.5 |  |
| Age |  |  | 0.441 |  |  | 0.969 |  |  | 0.820 |  |  | 0.424 |  |  | 0.150 |  |  | 0.941 |
| <60 | 116 | 53.4 |  |  | 44.8 |  |  | 34.5 |  |  | 25.9 |  |  | 16.4 |  |  | 16.4 |  |
| ≥60 | 162 | 48.8 |  |  | 45.1 |  |  | 35.8 |  |  | 30.2 |  |  | 23.5 |  |  | 16.0 |  |
| Smoking |  |  | 0.268 |  |  | 0.340 |  |  | 0.232 |  |  | 0.117 |  |  | 0.159 |  |  | 0.122 |
| No | 162 | 53.7 |  |  | 47.5 |  |  | 38.3 |  |  | 32.1 |  |  | 23.5 |  |  | 19.1 |  |
| Yes | 115 | 47.0 |  |  | 41.7 |  |  | 31.3 |  |  | 23.5 |  |  | 16.5 |  |  | 12.2 |  |
| Differentiation | |  | 0.125 |  |  | 0.171 |  |  | 0.140 |  |  | 0.615 |  |  | 0.384 |  |  | 0.478 |
| Well | 7 | 14.3 |  |  | 14.3 |  |  | 0 |  |  | 14.3 |  |  | 0 |  |  | 0 |  |
| Moderate | 156 | 53.2 |  |  | 48.1 |  |  | 36.5 |  |  | 27.6 |  |  | 20.5 |  |  | 16.0 |  |
| Poor | 115 | 49.6 |  |  | 42.6 |  |  | 35.7 |  |  | 30.4 |  |  | 21.7 |  |  | 17.4 |  |
| Invasive depth | |  | 0.462 |  |  | 0.441 |  |  | 0.920 |  |  | 0.840 |  |  | 0.937 |  |  | 0.836 |
| I | 89 | 53.9 |  |  | 48.3 |  |  | 34.8 |  |  | 29.2 |  |  | 20.2 |  |  | 16.9 |  |
| II | 189 | 49.2 |  |  | 43.4 |  |  | 35.4 |  |  | 28.0 |  |  | 20.6 |  |  | 33.7 |  |
| Vessel involvement | |  | 0.331 |  |  | 0.519 |  |  | 0.512 |  |  | 0.866 |  |  | 0.861 |  |  | 0.754 |
| No | 227 | 49.3 |  |  | 44.0 |  |  | 34.4 |  |  | 28.6 |  |  | 20.7 |  |  | 15.9 |  |
| Yes | 51 | 56.9 |  |  | 49.0 |  |  | 39.2 |  |  | 27.5 |  |  | 19.6 |  |  | 17.6 |  |
| Nerve involvement | |  | 0.995 |  |  | 0.821 |  |  | 0.939 |  |  | 0.407 |  |  | 0.316 |  |  | 0.763 |
| No | 205 | 50.7 |  |  | 45.4 |  |  | 35.1 |  |  | 29.8 |  |  | 22.0 |  |  | 16.6 |  |
| Yes | 73 | 50.7 |  |  | 43.8 |  |  | 35.6 |  |  | 24.7 |  |  | 16.4 |  |  | 15.1 |  |
| Lymph node metastasis | |  | 0.711 |  |  | 0.664 |  |  | 0.779 |  |  | 0.633 |  |  | 0.500 |  |  | 0.863 |
| No | 145 | 49.7 |  |  | 46.2 |  |  | 34.5 |  |  | 29.7 |  |  | 22.1 |  |  | 16.6 |  |
| Yes | 133 | 51.9 |  |  | 43.6 |  |  | 36.1 |  |  | 27.1 |  |  | 18.8 |  |  | 15.8 |  |
| Tumor site |  |  | 0.591 |  |  | 0.899 |  |  | 0.158 |  |  | 0.164 |  |  | 0.614 |  |  | 0.915 |
| Upper | 13 | 61.5 |  |  | 46.2 |  |  | 23.1 |  |  | 23.1 |  |  | 15.4 |  |  | 15.4 |  |
| Middle | 131 | 48.1 |  |  | 43.5 |  |  | 30.5 |  |  | 23.7 |  |  | 18.3 |  |  | 16.0 |  |
| Low | 123 | 52.0 |  |  | 46.3 |  |  | 40.7 |  |  | 34.1 |  |  | 22.8 |  |  | 17.9 |  |
| Clinical stage | |  | 0.201 |  |  | 0.383 |  |  | 0.064 |  |  | 0.309 |  |  | 0.892 |  |  | 0.691 |
| I-II | 168 | 47.6 |  |  | 42.9 |  |  | 30.9 |  |  | 26.2 |  |  | 20.2 |  |  | 15.5 |  |
| III-Iva | 110 | 55.5 |  |  | 48.2 |  |  | 41.8 |  |  | 31.8 |  |  | 20.9 |  |  | 17.3 |  |
| sPDL1 ≥1% | |  | 0.004 |  |  | 0.003 |  |  | <0.001 |  |  | <0.001 |  |  | 0.001 |  |  | 0.010 |
| No | 162 | 58.0 |  |  | 52.5 |  |  | 44.4 |  |  | 37.7 |  |  | 27.2 |  |  | 21.0 |  |
| Yes | 116 | 40.5 |  |  | 34.5 |  |  | 22.4 |  |  | 15.5 |  |  | 11.2 |  |  | 9.5 |  |
| sPDL1 ≥5% | |  | 0.041 |  |  | 0.044 |  |  | 0.002 |  |  | 0.001 |  |  | 0.006 |  |  | 0.034 |
| No | 171 | 55.6 |  |  | 49.7 |  |  | 42.1 |  |  | 35.7 |  |  | 25.7 |  |  | 19.9 |  |
| Yes | 107 | 43.0 |  |  | 37.3 |  |  | 24.3 |  |  | 16.8 |  |  | 12.1 |  |  | 10.3 |  |
| sPDL1 ≥10% | |  | 0.467 |  |  | 0.824 |  |  | 0.956 |  |  | 0.414 |  |  | 0.737 |  |  | 0.986 |
| No | 235 | 49.8 |  |  | 44.7 |  |  | 35.3 |  |  | 29.4 |  |  | 20.9 |  |  | 16.2 |  |
| Yes | 43 | 55.8 |  |  | 46.5 |  |  | 34.9 |  |  | 23.3 |  |  | 18.6 |  |  | 16.3 |  |
| sPDL1 ≥20% | |  | 0.570 |  |  | 0.969 |  |  | 0.778 |  |  | 0.472 |  |  | 0.800 |  |  | 0.927 |
| No | 236 | 50.0 |  |  | 44.9 |  |  | 35.6 |  |  | 29.2 |  |  | 20.8 |  |  | 16.1 |  |
| Yes | 42 | 54.8 |  |  | 45.2 |  |  | 33.3 |  |  | 23.8 |  |  | 19.0 |  |  | 16.7 |  |
| sPDL1 ≥30% | |  | 0.612 |  |  | 0.705 |  |  | 0.750 |  |  | 0.916 |  |  | 0.608 |  |  | 0.487 |
| No | 249 | 50.2 |  |  | 44.6 |  |  | 34.9 |  |  | 28.5 |  |  | 20.1 |  |  | 15.7 |  |
| Yes | 29 | 55.2 |  |  | 48.3 |  |  | 37.9 |  |  | 27.6 |  |  | 24.1 |  |  | 20.7 |  |
| sPDL1 ≥50% | |  | 0.162 |  |  | 0.154 |  |  | 0.399 |  |  | 0.633 |  |  | 0.429 |  |  | 0.472 |
| No | 260 | 49.6 |  |  | 43.8 |  |  | 34.6 |  |  | 28.1 |  |  | 20.0 |  |  | 15.8 |  |
| Yes | 18 | 66.7 |  |  | 61.1 |  |  | 44.4 |  |  | 33.3 |  |  | 27.8 |  |  | 22.2 |  |
| Invasive depth I, tumor invasion confined to muscularis; Invasive depth II, tumor invasion beyond the muscularis | | | | | | | | | | | | | | | | | | |
| tPDL1, Tumoral PDL1 expression; sPDL1, Stromal PDL1 expression. | | | | | | | | | | | | | | | | | | |

| Supplemental table 2 Correlation of PD-L1 expression with clinicopathological features in ESCC patients with Stage I-II disease | | | | | | | | | | | | | | | | | | |
| --- | --- | --- | --- | --- | --- | --- | --- | --- | --- | --- | --- | --- | --- | --- | --- | --- | --- | --- |
|  |  | tPDL1 ≥1% | |  | tPDL1 ≥5% | |  | tPDL1≥10% | |  | tPDL1 ≥20% | |  | tPDL1 ≥30% | |  | tPDL1 ≥50% | |
|  | N | % | *P* value |  | % | *P* value |  | % | *P* value |  | % | *P* value |  | % | *P* value |  | % | *P* value |
| Sex |  |  | 0.310 |  |  | 0.249 |  |  | 0.244 |  |  | 0.943 |  |  | 0.665 |  |  | 0.759 |
| Female | 35 | 40.0 |  |  | 34.3 |  |  | 22.9 |  |  | 25.7 |  |  | 22.9 |  |  | 17.1 |  |
| Male | 133 | 49.6 |  |  | 45.1 |  |  | 33.1 |  |  | 11.3 |  |  | 19.5 |  |  | 15.0 |  |
| Age |  |  | 0.266 |  |  | 0.644 |  |  | 0.947 |  |  | 0.653 |  |  | 0.311 |  |  | 0.858 |
| <60 | 62 | 53.2 |  |  | 45.2 |  |  | 30.6 |  |  | 24.2 |  |  | 16.1 |  |  | 16.1 |  |
| ≥60 | 106 | 44.3 |  |  | 41.5 |  |  | 31.1 |  |  | 27.4 |  |  | 22.6 |  |  | 15.1 |  |
| Smoking |  |  | 0.451 |  |  | 0.850 |  |  | 0.792 |  |  | 0.264 |  |  | 0.291 |  |  | 0.399 |
| No | 110 | 50.0 |  |  | 43.6 |  |  | 31.8 |  |  | 29.1 |  |  | 22.7 |  |  | 17.3 |  |
| Yes | 57 | 43.9 |  |  | 42.1 |  |  | 29.8 |  |  | 21.1 |  |  | 15.8 |  |  | 12.2 |  |
| Differentiation | |  | 0.373 |  |  | 0.206 |  |  | 0.120 |  |  | 0.851 |  |  | 0.450 |  |  | 0.533 |
| Well | 4 | 25.0 |  |  | 25.0 |  |  | 0 |  |  | 25.0 |  |  | 0.0 |  |  | 0.0 |  |
| Moderate | 97 | 51.5 |  |  | 48.5 |  |  | 35.1 |  |  | 27.8 |  |  | 22.7 |  |  | 17.5 |  |
| Poor | 67 | 43.3 |  |  | 35.8 |  |  | 26.9 |  |  | 23.9 |  |  | 17.9 |  |  | 13.4 |  |
| Invasive depth | |  | 0.546 |  |  | 0.562 |  |  | 0.836 |  |  | 0.854 |  |  | 0.540 |  |  | 0.768 |
| I | 82 | 50.0 |  |  | 45.1 |  |  | 31.7 |  |  | 26.8 |  |  | 18.3 |  |  | 14.6 |  |
| II | 86 | 45.3 |  |  | 40.7 |  |  | 30.2 |  |  | 25.6 |  |  | 22.1 |  |  | 16.3 |  |
| Vessel involvement | |  | 0.439 |  |  | 0.653 |  |  | 0.785 |  |  | 0.787 |  |  | 0.861 |  |  | 0.797 |
| No | 157 | 48.4 |  |  | 43.3 |  |  | 31.2 |  |  | 26.8 |  |  | 20.4 |  |  | 15.3 |  |
| Yes | 11 | 36.3 |  |  | 36.4 |  |  | 27.3 |  |  | 18.2 |  |  | 18.2 |  |  | 18.2 |  |
| Nerve involvement | |  | 0.483 |  |  | 0.358 |  |  | 0.264 |  |  | 0.158 |  |  | 0.105 |  |  | 0.206 |
| No | 137 | 48.9 |  |  | 44.5 |  |  | 32.8 |  |  | 28.5 |  |  | 22.6 |  |  | 17.5 |  |
| Yes | 31 | 41.9 |  |  | 35.5 |  |  | 22.6 |  |  | 16.1 |  |  | 9.7 |  |  | 6.5 |  |
| Lymph node metastasis | |  | 0.505 |  |  | 0.104 |  |  | 0.077 |  |  | 0.040 |  |  | 0.075 |  |  | 0.258 |
| No | 135 | 48.9 |  |  | 45.9 |  |  | 34.1 |  |  | 29.6 |  |  | 23.0 |  |  | 17.0 |  |
| Yes | 33 | 42.4 |  |  | 30.3 |  |  | 18.2 |  |  | 12.1 |  |  | 9.1 |  |  | 9.1 |  |
| Tumor site |  |  | 0.247 |  |  | 0.504 |  |  | 0.401 |  |  | 0.221 |  |  | 0.396 |  |  | 0.682 |
| Upper | 8 | 75.0 |  |  | 62.5 |  |  | 37.5 |  |  | 37.5 |  |  | 25.0 |  |  | 25.0 |  |
| Middle | 83 | 44.6 |  |  | 41.0 |  |  | 25.3 |  |  | 20.5 |  |  | 15.7 |  |  | 14.5 |  |
| Low | 66 | 47.0 |  |  | 42.4 |  |  | 34.8 |  |  | 31.8 |  |  | 24.2 |  |  | 18.2 |  |
| sPDL1 ≥1% |  |  | 0.015 |  |  | 0.019 |  |  | 0.001 |  |  | <0.001 |  |  | <0.001 |  |  | 0.010 |
| No | 97 | 55.7 |  |  | 50.5 |  |  | 41.2 |  |  | 37.1 |  |  | 29.9 |  |  | 21.7 |  |
| Yes | 71 | 36.6 |  |  | 32.4 |  |  | 16.9 |  |  | 11.3 |  |  | 7.0 |  |  | 7.0 |  |
| sPDL1 ≥5% |  |  | 0.116 |  |  | 0.120 |  |  | 0.005 |  |  | 0.001 |  |  | 0.001 |  |  | 0.027 |
| No | 103 | 52.4 |  |  | 47.6 |  |  | 38.8 |  |  | 35.0 |  |  | 28.2 |  |  | 20.4 |  |
| Yes | 65 | 40.0 |  |  | 35.4 |  |  | 18.5 |  |  | 12.3 |  |  | 7.2 |  |  | 7.7 |  |
| sPDL1 ≥10% | |  | 0.263 |  |  | 0.712 |  |  | 0.982 |  |  | 0.380 |  |  | 0.503 |  |  | 0.989 |
| No | 142 | 45.8 |  |  | 42.3 |  |  | 31.0 |  |  | 27.5 |  |  | 21.1 |  |  | 15.5 |  |
| Yes | 26 | 57.7 |  |  | 46.2 |  |  | 30.8 |  |  | 19.2 |  |  | 15.4 |  |  | 15.4 |  |
| sPDL1 ≥20% | |  | 0.263 |  |  | 0.712 |  |  | 0.982 |  |  | 0.380 |  |  | 0.503 |  |  | 0.989 |
| No | 142 | 45.8 |  |  | 42.2 |  |  | 31.0 |  |  | 27.5 |  |  | 21.1 |  |  | 15.5 |  |
| Yes | 26 | 57.7 |  |  | 46.2 |  |  | 30.8 |  |  | 19.2 |  |  | 15.4 |  |  | 15.4 |  |
| sPDL1 ≥30% | |  | 0.831 |  |  | 0.719 |  |  | 0.758 |  |  | 0.331 |  |  | 0.929 |  |  | 0.883 |
| No | 150 | 47.3 |  |  | 43.3 |  |  | 31.3 |  |  | 27.3 |  |  | 20.7 |  |  | 15.3 |  |
| Yes | 18 | 50.0 |  |  | 38.9 |  |  | 27.8 |  |  | 16.7 |  |  | 16.7 |  |  | 16.7 |  |
| sPDL1 ≥50% | |  | 0.634 |  |  | 0.857 |  |  | 0.785 |  |  | 0.787 |  |  | 0.861 |  |  | 0.797 |
| No | 157 | 47.1 |  |  | 42.7 |  |  | 31.2 |  |  | 26.8 |  |  | 20.4 |  |  | 15.3 |  |
| Yes | 11 | 54.5 |  |  | 45.5 |  |  | 27.3 |  |  | 18.2 |  |  | 18.2 |  |  | 18.2 |  |
| Invasive depth I, tumor invasion confined to muscularis; Invasive depth II, tumor invasion beyond the muscularis | | | | | | | | | | | | | | | | | | |
| tPDL1, Tumoral PDL1 expression; sPDL1, Stromal PDL1 expression. | | | | | | | | | | | | | | | | | | |

| Supplemental table 3 Correlation of PD-L1 expression with clinicopathological features in ESCC patients with Stage III-IV disease | | | | | | | | | | | | | | | | | | |
| --- | --- | --- | --- | --- | --- | --- | --- | --- | --- | --- | --- | --- | --- | --- | --- | --- | --- | --- |
|  |  | tPDL1 ≥1% | |  | tPDL1 ≥5% | |  | tPDL1≥10% | |  | tPDL1 ≥20% | |  | tPDL1 ≥30% | |  | tPDL1 ≥50% | |
|  | N | % | *P* value |  | % | *P* value |  | % | *P* value |  | % | *P* value |  | % | *P* value |  | % | *P* value |
| Sex |  |  | 0.949 |  |  | 0.656 |  |  | 0.367 |  |  | 0.733 |  |  | 0.184 |  |  | 0.355 |
| Female | 11 | 54.5 |  |  | 54.5 |  |  | 54.5 |  |  | 36.4 |  |  | 36.4 |  |  | 27.3 |  |
| Male | 99 | 55.6 |  |  | 47.5 |  |  | 40.4 |  |  | 31.3 |  |  | 19.2 |  |  | 16.2 |  |
| Age |  |  | 0.717 |  |  | 0.441 |  |  | 0.541 |  |  | 0.372 |  |  | 0.283 |  |  | 0.869 |
| <60 | 54 | 53.7 |  |  | 44.4 |  |  | 38.9 |  |  | 27.8 |  |  | 16.7 |  |  | 16.7 |  |
| ≥60 | 56 | 57.1 |  |  | 51.8 |  |  | 44.6 |  |  | 35.7 |  |  | 25.0 |  |  | 17.9 |  |
| Smoking |  |  | 0.224 |  |  | 0.132 |  |  | 0.042 |  |  | 0.157 |  |  | 0.318 |  |  | 0.127 |
| No | 52 | 61.5 |  |  | 55.8 |  |  | 51.9 |  |  | 38.5 |  |  | 25.0 |  |  | 23.1 |  |
| Yes | 58 | 50.0 |  |  | 41.4 |  |  | 32.8 |  |  | 25.9 |  |  | 17.2 |  |  | 12.1 |  |
| Differentiation | |  | 0.142 |  |  | 0.213 |  |  | 0.214 |  |  | 0.189 |  |  | 0.292 |  |  | 0.322 |
| Well | 3 | 0 |  |  | 0 |  |  | 0 |  |  | 0 |  |  | 0 |  |  | 0 |  |
| Moderate | 59 | 55.9 |  |  | 57.5 |  |  | 39.0 |  |  | 27.1 |  |  | 16.9 |  |  | 13.6 |  |
| Poor | 48 | 58.3 |  |  | 52.1 |  |  | 47.9 |  |  | 39.6 |  |  | 27.1 |  |  | 22.9 |  |
| Invasive depth | |  | 0.014 |  |  | 0.040 |  |  | 0.101 |  |  | 0.137 |  |  | 0.140 |  |  | 0.064 |
| I | 7 | 100 |  |  | 85.7 |  |  | 71.4 |  |  | 57.1 |  |  | 42.9 |  |  | 42.9 |  |
| II | 103 | 52.4 |  |  | 45.6 |  |  | 39.8 |  |  | 30.1 |  |  | 19.4 |  |  | 15.5 |  |
| Vessel involvement | |  | 0.261 |  |  | 0.493 |  |  | 0.913 |  |  | 0.757 |  |  | 0.859 |  |  | 0.962 |
| No | 70 | 51.4 |  |  | 45.7 |  |  | 41.4 |  |  | 32.9 |  |  | 21.4 |  |  | 17.1 |  |
| Yes | 40 | 62.5 |  |  | 52.5 |  |  | 42.5 |  |  | 30.0 |  |  | 20.0 |  |  | 17.5 |  |
| Nerve involvement | |  | 0.779 |  |  | 0.764 |  |  | 0.568 |  |  | 0.878 |  |  | 0.916 |  |  | 0.365 |
| No | 68 | 54.4 |  |  | 47.1 |  |  | 39.7 |  |  | 32.3 |  |  | 20.6 |  |  | 14.7 |  |
| Yes | 42 | 57.1 |  |  | 50.0 |  |  | 45.2 |  |  | 31.0 |  |  | 21.4 |  |  | 21.4 |  |
| Lymph node metastasis | |  | 0.762 |  |  | 0.904 |  |  | 0.903 |  |  | 0.897 |  |  | 0.374 |  |  | 0.523 |
| No | 10 | 60.0 |  |  | 50.0 |  |  | 40.0 |  |  | 30.0 |  |  | 10.0 |  |  | 10.0 |  |
| Yes | 100 | 55.0 |  |  | 48.0 |  |  | 42.0 |  |  | 32.0 |  |  | 22.0 |  |  | 18.0 |  |
| Tumor site |  |  | 0.721 |  |  | 0.415 |  |  | 0.044 |  |  | 0.207 |  |  | 0.487 |  |  | 0.571 |
| Upper | 5 | 40.0 |  |  | 20.0 |  |  | 0 |  |  | 0 |  |  | 0 |  |  | 0 |  |
| Middle | 48 | 94.2 |  |  | 47.9 |  |  | 39.6 |  |  | 29.2 |  |  | 22.9 |  |  | 18.8 |  |
| Low | 57 | 57.9 |  |  | 50.9 |  |  | 47.4 |  |  | 36.8 |  |  | 21.1 |  |  | 17.5 |  |
| sPDL1 ≥1% |  |  | 0.123 |  |  | 0.069 |  |  | 0.058 |  |  | 0.072 |  |  | 0.502 |  |  | 0.363 |
| No | 65 | 61.5 |  |  | 55.4 |  |  | 49.2 |  |  | 38.5 |  |  | 23.1 |  |  | 20.0 |  |
| Yes | 45 | 46.7 |  |  | 37.8 |  |  | 31.1 |  |  | 22.2 |  |  | 17.8 |  |  | 13.3 |  |
| sPDL1 ≥5% |  |  | 0.194 |  |  | 0.204 |  |  | 0.156 |  |  | 0.156 |  |  | 0.706 |  |  | 0.515 |
| No | 68 | 60.3 |  |  | 52.9 |  |  | 47.1 |  |  | 36.8 |  |  | 22.1 |  |  | 19.1 |  |
| Yes | 42 | 47.6 |  |  | 40.5 |  |  | 33.3 |  |  | 23.8 |  |  | 19.0 |  |  | 14.3 |  |
| sPDL1 ≥10% | |  | 0.821 |  |  | 0.920 |  |  | 0.953 |  |  | 0.817 |  |  | 0.773 |  |  | 0.965 |
| No | 93 | 55.9 |  |  | 48.4 |  |  | 41.9 |  |  | 32.3 |  |  | 20.4 |  |  | 17.2 |  |
| Yes | 17 | 52.9 |  |  | 47.1 |  |  | 41.2 |  |  | 29.4 |  |  | 23.5 |  |  | 17.6 |  |
| sPDL1 ≥20% | |  | 0.635 |  |  | 0.701 |  |  | 0.705 |  |  | 0.958 |  |  | 0.663 |  |  | 0.866 |
| No | 94 | 56.4 |  |  | 48.9 |  |  | 42.6 |  |  | 31.9 |  |  | 20.2 |  |  | 17.0 |  |
| Yes | 16 | 50.0 |  |  | 43.8 |  |  | 37.5 |  |  | 31.3 |  |  | 25.0 |  |  | 18.8 |  |
| sPDL1 ≥30% | |  | 0.565 |  |  | 0.280 |  |  | 0.367 |  |  | 0.306 |  |  | 0.184 |  |  | 0.355 |
| No | 99 | 54.5 |  |  | 46.5 |  |  | 40.4 |  |  | 30.3 |  |  | 19.2 |  |  | 16.2 |  |
| Yes | 11 | 63.6 |  |  | 63.6 |  |  | 54.5 |  |  | 45.5 |  |  | 36.4 |  |  | 27.3 |  |
| sPDL1 ≥50% | |  | 0.096 |  |  | 0.096 |  |  | 0.101 |  |  | 0.137 |  |  | 0.140 |  |  | 0.414 |
| No | 103 | 53.4 |  |  | 45.6 |  |  | 39.8 |  |  | 30.1 |  |  | 19.4 |  |  | 16.5 |  |
| Yes | 7 | 85.7 |  |  | 85.7 |  |  | 71.4 |  |  | 57.1 |  |  | 42.9 |  |  | 28.6 |  |
| Invasive depth I, tumor invasion confined to muscularis; Invasive depth II, tumor invasion beyond the muscularis | | | | | | | | | | | | | | | | | | |
| tPDL1, Tumoral PDL1 expression; sPDL1, Stromal PDL1 expression. | | | | | | | | | | | | | | | | | | |

| Supplemental table 4 Correlation of PD-L1 expression with clinicopathological features in ESCC patients without lympha node metastasis | | | | | | | | | | | | | | | | | | |
| --- | --- | --- | --- | --- | --- | --- | --- | --- | --- | --- | --- | --- | --- | --- | --- | --- | --- | --- |
|  |  | tPDL1 ≥1% | |  | tPDL1 ≥5% | |  | tPDL1≥10% | |  | tPDL1 ≥20% | |  | tPDL1 ≥30% | |  | tPDL1 ≥50% | |
|  | N | % | *P* value |  | % | *P* value |  | % | *P* value |  | % | *P* value |  | % | *P* value |  | % | *P* value |
| Sex |  |  | 0.868 |  |  | 0.560 |  |  | 0.382 |  |  | 0.856 |  |  | 0.423 |  |  | 0.503 |
| Female | 29 | 48.3 |  |  | 41.4 |  |  | 27.6 |  |  | 31.0 |  |  | 27.6 |  |  | 20.7 |  |
| Male | 116 | 50.0 |  |  | 47.4 |  |  | 36.2 |  |  | 29.3 |  |  | 20.7 |  |  | 15.5 |  |
| Age |  |  | 0.814 |  |  | 0.585 |  |  | 0.562 |  |  | 0.419 |  |  | 0.344 |  |  | 0.794 |
| <60 | 51 | 51.0 |  |  | 43.1 |  |  | 31.4 |  |  | 25.5 |  |  | 17.6 |  |  | 17.6 |  |
| ≥60 | 94 | 48.9 |  |  | 47.9 |  |  | 36.2 |  |  | 31.9 |  |  | 24.5 |  |  | 16.0 |  |
| Smoking |  |  | 0.379 |  |  | 0.778 |  |  | 0.708 |  |  | 0.075 |  |  | 0.222 |  |  | 0.307 |
| No | 95 | 52.6 |  |  | 47.4 |  |  | 35.8 |  |  | 34.7 |  |  | 25.3 |  |  | 18.9 |  |
| Yes | 49 | 44.9 |  |  | 44.9 |  |  | 32.7 |  |  | 20.4 |  |  | 16.3 |  |  | 12.2 |  |
| Differentiation | |  | 0.213 |  |  | 0.137 |  |  | 0.198 |  |  | 0.801 |  |  | 0.297 |  |  | 0.334 |
| Well | 4 | 25.0 |  |  | 25.0 |  |  | 0.0 |  |  | 25.0 |  |  | 0.0 |  |  | 0.0 |  |
| Moderate | 85 | 55.3 |  |  | 52.9 |  |  | 38.8 |  |  | 31.8 |  |  | 25.9 |  |  | 20.0 |  |
| Poor | 56 | 42.9 |  |  | 37.5 |  |  | 30.4 |  |  | 26.8 |  |  | 17.9 |  |  | 12.5 |  |
| Invasive depth | |  | 0.362 |  |  | 0.198 |  |  | 0.733 |  |  | 0.737 |  |  | 0.851 |  |  | 0.965 |
| I | 61 | 54.1 |  |  | 52.5 |  |  | 36.1 |  |  | 31.1 |  |  | 21.3 |  |  | 16.4 |  |
| II | 84 | 46.4 |  |  | 41.7 |  |  | 33.3 |  |  | 28.6 |  |  | 22.6 |  |  | 16.7 |  |
| Vessel involvement | |  | 0.455 |  |  | 0.342 |  |  | 0.342 |  |  | 0.617 |  |  | 0.837 |  |  | 0.508 |
| No | 137 | 48.9 |  |  | 45.3 |  |  | 33.6 |  |  | 29.2 |  |  | 21.9 |  |  | 16.1 |  |
| Yes | 8 | 62.5 |  |  | 62.5 |  |  | 50.0 |  |  | 37.5 |  |  | 25.0 |  |  | 25.0 |  |
| Nerve involvement | |  | 0.561 |  |  | 0.318 |  |  | 0.190 |  |  | 0.102 |  |  | 0.089 |  |  | 0.118 |
| No | 116 | 50.9 |  |  | 48.3 |  |  | 37.1 |  |  | 32.8 |  |  | 25.0 |  |  | 19.0 |  |
| Yes | 29 | 44.8 |  |  | 37.9 |  |  | 24.1 |  |  | 17.2 |  |  | 10.3 |  |  | 6.9 |  |
| Tumor site |  |  | 0.583 |  |  | 0.775 |  |  | 0.354 |  |  | 0.164 |  |  | 0.129 |  |  | 0.349 |
| Upper | 7 | 57.1 |  |  | 42.9 |  |  | 28.6 |  |  | 28.6 |  |  | 28.6 |  |  | 28.6 |  |
| Middle | 73 | 45.2 |  |  | 43.8 |  |  | 28.8 |  |  | 23.3 |  |  | 15.1 |  |  | 13.7 |  |
| Low | 54 | 53.7 |  |  | 50.0 |  |  | 40.7 |  |  | 38.9 |  |  | 29.6 |  |  | 22.2 |  |
| Clinical stage | |  | 0.498 |  |  | 0.803 |  |  | 0.704 |  |  | 0.980 |  |  | 0.340 |  |  | 0.891 |
| I-II | 135 | 48.9 |  |  | 45.9 |  |  | 34.1 |  |  | 29.6 |  |  | 23.0 |  |  | 17.0 |  |
| III-Iva | 10 | 60.0 |  |  | 50.0 |  |  | 40.0 |  |  | 30.0 |  |  | 10.0 |  |  | 10.0 |  |
| sPDL1 ≥1% |  |  | 0.261 |  |  | 0.139 |  |  | 0.002 |  |  | 0.001 |  |  | 0.007 |  |  | 0.116 |
| No | 88 | 53.4 |  |  | 51.1 |  |  | 44.3 |  |  | 39.8 |  |  | 29.5 |  |  | 20.5 |  |
| Yes | 57 | 43.9 |  |  | 38.6 |  |  | 19.3 |  |  | 14.0 |  |  | 10.5 |  |  | 10.5 |  |
| sPDL1 ≥5% |  |  | 0.533 |  |  | 0.309 |  |  | 0.006 |  |  | 0.003 |  |  | 0.014 |  |  | 0.174 |
| No | 91 | 51.6 |  |  | 54.9 |  |  | 42.9 |  |  | 38.5 |  |  | 28.6 |  |  | 19.8 |  |
| Yes | 54 | 46.3 |  |  | 40.7 |  |  | 20.4 |  |  | 14.8 |  |  | 11.1 |  |  | 11.1 |  |
| sPDL1 ≥10% | |  | 0.168 |  |  | 0.683 |  |  | 0.897 |  |  | 0.585 |  |  | 0.873 |  |  | 0.537 |
| No | 121 | 47.1 |  |  | 45.5 |  |  | 34.7 |  |  | 30.6 |  |  | 22.3 |  |  | 15.7 |  |
| Yes | 24 | 62.5 |  |  | 50.0 |  |  | 33.3 |  |  | 25.0 |  |  | 20.8 |  |  | 20.8 |  |
| sPDL1 ≥20% | |  | 0.168 |  |  | 0.683 |  |  | 0.897 |  |  | 0.585 |  |  | 0.873 |  |  | 0.537 |
| No | 121 | 47.1 |  |  | 45.5 |  |  | 34.7 |  |  | 30.6 |  |  | 22.3 |  |  | 15.7 |  |
| Yes | 24 | 62.5 |  |  | 50.0 |  |  | 33.3 |  |  | 25.0 |  |  | 20.8 |  |  | 20.8 |  |
| sPDL1 ≥30% | |  | 0.421 |  |  | 0.940 |  |  | 0.940 |  |  | 0.556 |  |  | 0.877 |  |  | 0.410 |
| No | 128 | 48.4 |  |  | 46.1 |  |  | 34.4 |  |  | 30.5 |  |  | 21.9 |  |  | 15.6 |  |
| Yes | 17 | 58.8 |  |  | 47.1 |  |  | 35.3 |  |  | 23.5 |  |  | 23.5 |  |  | 23.5 |  |
| sPDL1 ≥50% | |  | 0.335 |  |  | 0.564 |  |  | 0.891 |  |  | 0.857 |  |  | 0.665 |  |  | 0.320 |
| No | 134 | 48.5 |  |  | 45.5 |  |  | 34.3 |  |  | 29.9 |  |  | 21.6 |  |  | 15.7 |  |
| Yes | 11 | 63.6 |  |  | 54.5 |  |  | 36.4 |  |  | 27.3 |  |  | 27.3 |  |  | 27.3 |  |
| Invasive depth I, tumor invasion confined to muscularis; Invasive depth II, tumor invasion beyond the muscularis | | | | | | | | | | | | | | | | | | |
| tPDL1, Tumoral PDL1 expression; sPDL1, Stromal PDL1 expression. | | | | | | | | | | | | | | | | | | |

| Supplemental table 5 Correlation of PD-L1 expression with clinicopathological features in ESCC patients with lympha node metastasis | | | | | | | | | | | | | | | | | | |
| --- | --- | --- | --- | --- | --- | --- | --- | --- | --- | --- | --- | --- | --- | --- | --- | --- | --- | --- |
|  |  | tPDL1 ≥1% | |  | tPDL1 ≥5% | |  | tPDL1≥10% | |  | tPDL1 ≥20% | |  | tPDL1 ≥30% | |  | tPDL1 ≥50% | |
|  | N | % | *P* value |  | % | *P* value |  | % | *P* value |  | % | *P* value |  | % | *P* value |  | % | *P* value |
| Sex |  |  | 0.143 |  |  | 0.459 |  |  | 0.942 |  |  | 0.725 |  |  | 0.593 |  |  | 0.822 |
| Female | 17 | 35.3 |  |  | 35.3 |  |  | 35.3 |  |  | 23.5 |  |  | 23.5 |  |  | 17.6 |  |
| Male | 116 | 54.3 |  |  | 44.8 |  |  | 36.2 |  |  | 27.6 |  |  | 18.1 |  |  | 15.5 |  |
| Age |  |  | 0.429 |  |  | 0.563 |  |  | 0.845 |  |  | 0.817 |  |  | 0.325 |  |  | 0.900 |
| <60 | 65 | 55.4 |  |  | 46.2 |  |  | 36.9 |  |  | 26.2 |  |  | 15.4 |  |  | 15.4 |  |
| ≥60 | 68 | 48.5 |  |  | 41.2 |  |  | 35.3 |  |  | 27.9 |  |  | 22.1 |  |  | 16.2 |  |
| Smoking |  |  | 0.437 |  |  | 0.331 |  |  | 0.168 |  |  | 0.736 |  |  | 0.533 |  |  | 0.250 |
| No | 112 | 55.2 |  |  | 47.8 |  |  | 41.8 |  |  | 28.3 |  |  | 20.9 |  |  | 19.4 |  |
| Yes | 21 | 48.5 |  |  | 39.4 |  |  | 30.3 |  |  | 25.8 |  |  | 16.7 |  |  | 12.1 |  |
| Differentiation | |  | 0.160 |  |  | 0.256 |  |  | 0.302 |  |  | 0.197 |  |  | 0.180 |  |  | 0.184 |
| Well | 3 | 0.0 |  |  | 0.0 |  |  | 0.0 |  |  | 0.0 |  |  | 0.0 |  |  | 0.0 |  |
| Moderate | 71 | 50.7 |  |  | 42.3 |  |  | 33.8 |  |  | 22.5 |  |  | 14.1 |  |  | 11.3 |  |
| Poor | 59 | 55.9 |  |  | 47.5 |  |  | 40.7 |  |  | 33.9 |  |  | 25.4 |  |  | 22.0 |  |
| Invasive depth | |  | 0.840 |  |  | 0.604 |  |  | 0.624 |  |  | 0.782 |  |  | 0.886 |  |  | 0.736 |
| I | 28 | 53.6 |  |  | 39.3 |  |  | 32.1 |  |  | 25.0 |  |  | 17.9 |  |  | 17.9 |  |
| II | 105 | 51.4 |  |  | 44.8 |  |  | 37.1 |  |  | 27.6 |  |  | 19.0 |  |  | 15.2 |  |
| Vessel involvement | |  | 0.530 |  |  | 0.641 |  |  | 0.853 |  |  | 0.790 |  |  | 0.969 |  |  | 0.915 |
| No | 90 | 50.0 |  |  | 42.2 |  |  | 35.6 |  |  | 27.8 |  |  | 18.9 |  |  | 15.6 |  |
| Yes | 43 | 55.8 |  |  | 46.5 |  |  | 37.2 |  |  | 25.6 |  |  | 18.6 |  |  | 16.3 |  |
| Nerve involvement | |  | 0.665 |  |  | 0.501 |  |  | 0.231 |  |  | 0.651 |  |  | 0.731 |  |  | 0.300 |
| No | 89 | 50.6 |  |  | 41.6 |  |  | 32.6 |  |  | 25.8 |  |  | 18.0 |  |  | 13.5 |  |
| Yes | 44 | 54.5 |  |  | 47.7 |  |  | 43.2 |  |  | 29.5 |  |  | 20.5 |  |  | 20.5 |  |
| Tumor site |  |  | 0.755 |  |  | 0.948 |  |  | 0.394 |  |  | 0.613 |  |  | 0.372 |  |  | 0.438 |
| Upper | 6 | 66.7 |  |  | 50.0 |  |  | 16.7 |  |  | 16.7 |  |  | 0.0 |  |  | 0.0 |  |
| Middle | 58 | 51.7 |  |  | 43.1 |  |  | 32.8 |  |  | 24.1 |  |  | 22.4 |  |  | 19.0 |  |
| Low | 69 | 50.7 |  |  | 43.5 |  |  | 40.6 |  |  | 30.4 |  |  | 17.4 |  |  | 14.5 |  |
| Clinical stage | |  | 0.210 |  |  | 0.075 |  |  | 0.013 |  |  | 0.026 |  |  | 0.100 |  |  | 0.224 |
| I-II | 33 | 42.4 |  |  | 30.3 |  |  | 18.2 |  |  | 12.1 |  |  | 9.1 |  |  | 9.1 |  |
| III-Iva | 100 | 55.0 |  |  | 48.0 |  |  | 4.2 |  |  | 32.0 |  |  | 22.0 |  |  | 18.0 |  |
| sPDL1 ≥1% |  |  | 0.003 |  |  | 0.007 |  |  | 0.022 |  |  | 0.019 |  |  | 0.068 |  |  | 0.039 |
| No | 74 | 63.5 |  |  | 54.1 |  |  | 44.6 |  |  | 35.1 |  |  | 24.3 |  |  | 21.6 |  |
| Yes | 59 | 37.3 |  |  | 30.5 |  |  | 25.4 |  |  | 16.9 |  |  | 11.9 |  |  | 8.5 |  |
| sPDL1 ≥5% |  |  | 0.021 |  |  | 0.068 |  |  | 0.128 |  |  | 0.083 |  |  | 0.179 |  |  | 0.102 |
| No | 80 | 60.0 |  |  | 50.0 |  |  | 41.3 |  |  | 32.5 |  |  | 22.5 |  |  | 20.0 |  |
| Yes | 53 | 39.6 |  |  | 34.0 |  |  | 28.3 |  |  | 18.9 |  |  | 13.2 |  |  | 9.4 |  |
| sPDL1 ≥10% | |  | 0.671 |  |  | 0.886 |  |  | 0.941 |  |  | 0.524 |  |  | 0.717 |  |  | 0.497 |
| No | 114 | 52.6 |  |  | 48.9 |  |  | 36.0 |  |  | 28.1 |  |  | 19.3 |  |  | 16.7 |  |
| Yes | 19 | 47.4 |  |  | 42.1 |  |  | 36.8 |  |  | 21.1 |  |  | 15.8 |  |  | 10.5 |  |
| sPDL1 ≥20% | |  | 0.497 |  |  | 0.664 |  |  | 0.793 |  |  | 0.619 |  |  | 0.804 |  |  | 0.558 |
| No | 115 | 53.0 |  |  | 44.3 |  |  | 36.5 |  |  | 27.8 |  |  | 19.1 |  |  | 16.5 |  |
| Yes | 18 | 44.4 |  |  | 38.9 |  |  | 33.3 |  |  | 22.2 |  |  | 16.7 |  |  | 11.1 |  |
| sPDL1 ≥30% | |  | 0.891 |  |  | 0.640 |  |  | 0.673 |  |  | 0.609 |  |  | 0.564 |  |  | 0.930 |
| No | 121 | 52.1 |  |  | 43.0 |  |  | 35.5 |  |  | 26.4 |  |  | 18.2 |  |  | 15.7 |  |
| Yes | 12 | 50.0 |  |  | 50.0 |  |  | 41.7 |  |  | 33.3 |  |  | 25.0 |  |  | 16.7 |  |
| sPDL1 ≥50% | |  | 0.288 |  |  | 0.127 |  |  | 0.233 |  |  | 0.334 |  |  | 0.496 |  |  | 0.911 |
| No | 126 | 50.8 |  |  | 42.1 |  |  | 34.9 |  |  | 26.2 |  |  | 18.3 |  |  | 15.9 |  |
| Yes | 7 | 71.4 |  |  | 71.4 |  |  | 57.1 |  |  | 42.9 |  |  | 28.6 |  |  | 14.3 |  |
| Invasive depth I, tumor invasion confined to muscularis; Invasive depth II, tumor invasion beyond the muscularis | | | | | | | | | | | | | | | | | | |
| tPDL1, Tumoral PDL1 expression; sPDL1, Stromal PDL1 expression. | | | | | | | | | | | | | | | | | | |

| Supplemental table 6 Kaplan–Meier survival analysis for disease free survival and esophageal cancer-specific survival | | | | | | | | | | | | | | | | | | | |
| --- | --- | --- | --- | --- | --- | --- | --- | --- | --- | --- | --- | --- | --- | --- | --- | --- | --- | --- | --- |
|  | All patients | | |  | Patients with Stage I-II disease | | |  | Patients with Stage III-IVa disease | | |  | Patients without lymph node metastasis | | |  | Patients with lymph node metastasis | | |
|  | N | DFS | OS |  | N | DFS | OS |  | N | DFS | OS |  | N | DFS | OS |  | N | DFS | OS |
| tPDL1 ≥1% | 137 | 0.110 | 0.068 |  | 88 | 0.039 | 0.025 |  | 49 | 0.247 | 0.292 |  | 73 | 0.026 | 0.019 |  | 64 | 0.973 | 0.864 |
|  | 141 |  |  |  | 80 |  |  |  | 61 |  |  |  | 72 |  |  |  | 69 |  |  |
| tPDL1 ≥5% | 153 | 0.047 | 0.031 |  | 96 | 0.007 | 0.003 |  | 57 | 0.599 | 0.747 |  | 78 | 0.024 | 0.015 |  | 75 | 0.996 | 0.943 |
|  | 125 |  |  |  | 72 |  |  |  | 53 |  |  |  | 67 |  |  |  | 58 |  |  |
| tPDL1 ≥10% | 180 | 0.039 | 0.036 |  | 116 | 0.003 | 0.003 |  | 64 | 0.220 | 0.272 |  | 95 | 0.015 | 0.016 |  | 85 | 0.622 | 0.610 |
|  | 98 |  |  |  | 52 |  |  |  | 66 |  |  |  | 50 |  |  |  | 48 |  |  |
| tPDL1 ≥20% | 199 | 0.011 | 0.010 |  | 124 | 0.002 | 0.003 |  | 75 | 0.313 | 0.266 |  | 102 | 0.003 | 0.007 |  | 97 | 0.881 | 0.675 |
|  | 79 |  |  |  | 44 |  |  |  | 35 |  |  |  | 43 |  |  |  | 36 |  |  |
| tPDL1 ≥30% | 221 | 0.002 | 0.002 |  | 134 | 0.002 | 0.005 |  | 87 | 0.151 | 0.081 |  | 113 | 0.005 | 0.011 |  | 108 | 0.254 | 0.126 |
|  | 57 |  |  |  | 34 |  |  |  | 23 |  |  |  | 32 |  |  |  | 25 |  |  |
| tPDL1 ≥50% | 233 | 0.050 | 0.039 |  | 142 | 0.056 | 0.091 |  | 91 | 0.202 | 0.108 |  | 121 | 0.086 | 0.139 |  | 112 | 0.291 | 0.138 |
|  | 45 |  |  |  | 26 |  |  |  | 19 |  |  |  | 24 |  |  |  | 21 |  |  |
| sPDL1 ≥1% | 162 | 0.498 | 0.680 |  | 97 | 0.101 | 0.104 |  | 65 | 0.447 | 0.157 |  | 88 | 0.109 | 0.130 |  | 74 | 0.099 | 0.051 |
|  | 116 |  |  |  | 71 |  |  |  | 45 |  |  |  | 57 |  |  |  | 59 |  |  |
| sPDL1 ≥5% | 171 | 0.614 | 0.773 |  | 103 | 0.207 | 0.207 |  | 68 | 0.465 | 0.190 |  | 91 | 0.101 | 0.118 |  | 80 | 0.114 | 0.073 |
|  | 107 |  |  |  | 65 |  |  |  | 42 |  |  |  | 54 |  |  |  | 53 |  |  |
| sPDL1 ≥10% | 235 | 0.206 | 0.132 |  | 142 | 0.340 | 0.248 |  | 93 | 0.496 | 0.221 |  | 121 | 0.349 | 0.221 |  | 114 | 0.513 | 0.412 |
|  | 43 |  |  |  | 26 |  |  |  | 17 |  |  |  | 24 |  |  |  | 19 |  |  |
| sPDL1 ≥20% | 236 | 0.264 | 0.166 |  | 142 | 0.340 | 0.248 |  | 94 | 0.813 | 0.368 |  | 121 | 0.349 | 0.221 |  | 115 | 0.780 | 0.591 |
|  | 42 |  |  |  | 26 |  |  |  | 16 |  |  |  | 24 |  |  |  | 18 |  |  |
| sPDL1 ≥30% | 249 | 0.256 | 0.264 |  | 150 | 0.369 | 0.444 |  | 99 | 0.601 | 0.367 |  | 128 | 0.428 | 0.542 |  | 121 | 0.654 | 0.416 |
|  | 29 |  |  |  | 18 |  |  |  | 11 |  |  |  | 17 |  |  |  | 12 |  |  |
| sPDL1 ≥50% | 260 | 0.422 | 0.400 |  | 157 | 0.628 | 0.742 |  | 103 | 0.484 | 0.248 |  | 134 | 0.822 | 0.993 |  | 126 | 0.622 | 0.353 |
|  | 18 |  |  |  | 11 |  |  |  | 7 |  |  |  | 11 |  |  |  | 7 |  |  |
| DFS, disease free survival; OS, overall survival. | | | | | | | | | | | | | | | | | | | |
| tPDL1, Tumoral PDL1 expression; sPDL1, Stromal PDL1 expression. | | | | | | | | | | | | | | | | | | | |
